# Gender differences in self‐reported family history of cancer: A review and secondary data analysis

**DOI:** 10.1002/cam4.3405

**Published:** 2020-08-24

**Authors:** Monika Sieverding, Anna Lisa Arbogast, Stephanie Zintel, Christian von Wagner

**Affiliations:** ^1^ Department of Psychology Ruprecht Karls University Heidelberg Heidelberg Germany; ^2^ Research Department of Behavioural Science and Health University College London London UK

**Keywords:** family history of cancer, gender difference, population‐based studies, self‐reports

## Abstract

**Background:**

Assessment of family history of cancer (FHC) mostly relies on self‐report. Our goal was to find out whether there is a systematic gender difference in self‐reported FHC.

**Methods:**

We identified nine population‐based studies which provided statistics of FHC in men and women (N_1_ = 404 541). Furthermore, we analyzed data (N_2_ = 167 154) from several iterations of the US‐based Health Information National Trends Survey (HINTS) and the National Health Interview Survey (NHIS). We calculated the proportion of positive FHC, odds ratios (OR M/F), 95% confidence intervals, and aggregated statistics. We additionally analyzed in‐depth questions about FHC from HINTS 5 Cycle 2.

**Results:**

In the reviewed studies the odds of men reporting a FHC were lower compared with the odds of women with an average OR of 0.84 [0.71; 1.00] across all studies and an OR of 0.75 [0.70; 0.80] for the six studies from the US and Europe. The gender gap was replicated in our own analyses of HINTS and NHIS with an average OR of 0.75 [0.71; 0.79]. In HINTS 5 Cycle 2 men described themselves as less familiar with their FHC and less confident answering questions regarding FHC. They were also less likely to discuss FHC with family members.

**Conclusions:**

Men— at least in the US and Europe—were consistently less likely to report FHC compared with women. Future research should investigate how the assessment of FHC can be improved to reduce these differences. Health care professionals should also consider the potential for biased reporting by gender when assessing FHC.

## Graphical abstract

A review of population‐based studies and a secondary data analysis of several iterations of two large US surveys (HINTS and NHIS) revealed a consistent gender gap in self‐reported family history of cancer. Men—at least in the US and Europe—are consistently less likely to report a family history of cancer.

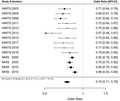

## 1 INTRODUCTION

A positive family history of cancer (FHC) is a risk factor for many cancer types.^1^, ^2^ A comprehensive FHC as part of a broader family health history is regarded as a central tool for risk assessment and prevention management.^2^, ^3^ The American Cancer Society recommends, that people with a FHC should be identified and motivated to participate in genetic counseling as well as earlier and/ or more intensive cancer screening.^4^ While currently cancer screening programs only use gender and age to define their target population, FHC reporting could become an important component in the way people would be invited to risk‐stratified screening iterations of established or future programs. In medical routine, FHC is usually assessed by self‐report of individuals, for example, in anamnesis interviews conducted by general practitioners or in a hospital.

There is accumulating evidence that men are less likely to report FHC compared with women.^5^, ^6^, ^7^, ^8^ Lower awareness of FHC among men can have serious consequences on their preventive behaviors like information seeking about cancer, or genetic counseling. A recent editorial comment on the profile of men's health came to the conclusion that “men need to care more about their own health.”^9^ However, an important prerequisite for taking care of one's health is the perception that one might be susceptible to a certain disease and that taking care is necessary and helpful.^10^ The knowledge that one or more first‐degree relatives did have cancer is one important factor for the self‐assessment that one could be at risk for getting cancer as well. Indeed, several analyses using data from the large US National Health Interview Survey (NHIS) identified clear gender differences in awareness and use of genetic counseling and testing.^11^, ^12^ Male sex was identified as a factor associated with lower awareness regarding cancer genetic testing^11^ and another recent analysis of NHIS 2015 data revealed that in the US men had made less use of genetic counseling than women.^12^

Up to now the evidence regarding gender differences in self‐reported FHC has not been systematically gathered and analyzed across studies. Moreover, several surveys present substantial gender differences in self‐reported FHC in their result tables without discussing them in the text.^5^, ^13^, ^14^, ^15^ The goal of our research was to find out whether there is a systematic gender difference in self‐reported FHC by reviewing and analyzing data from population‐based community samples in which adult men and women were asked about their FHC.

## 2 METHODS

### 2.1 Overview

First, we reviewed published surveys from different countries in which FHC was assessed in men and women. We then conducted gender comparisons of self‐reported FHC in original data from two recurring nationally representative surveys which were conducted in the US.

### 2.2 Review

We conducted electronic searches in the databases PubMed and Web of Science for population‐based surveys with adults. As search terms “family history of cancer” was used for all fields, as well as “questionnaire or survey or interview or cross sectional” and “women and men or female* and male* or gender or sex.” We explicitly excluded results with “case report or clinical study or randomized trial or case control” from the search. No search time frame was used. Only quantitative cross‐sectional surveys that were published in English or German language and provided exact frequency data about self‐reported FHC for any cancer for men and women were included. Studies on specific subgroups of the population such as students or smokers were excluded, likewise publications in languages other than English or German. Longitudinal studies were only included if they included baseline assessments for the relevant variables. The electronic searches were supplemented by manual searches in relevant studies and bibliographies that were identified in prior research by MS and CvW. Electronic searches were done on Nov 20, 2019 (see Supplemental material S1 for further information on the search strategy and S2 and Figure S1 for study selection).

To determine the prevalence of self‐reported FHC, we extracted the absolute numbers and percentage of men and women who reported a positive FHC from the result tables of the included publications. We calculated odds ratios (the odds of men reporting a positive FHC compared with the odds of women) and 95% confidence intervals for all studies.

In order to estimate a mean gender effect across studies, we used the metafor package (version 2.1.0) for R.^19^ Random‐effects models were fitted to the data using restricted maximum‐likelihood estimation.

### 2.3 Analyses of original data

To find out whether there is a systematic gender difference in self‐reported FHC, we furthermore analyzed original data. Two US‐based cross‐sectional cancer‐related population surveys, the Health Information National Trends Survey (HINTS)^20^ and the National Health Interview Survey (NHIS)^21^ which have been repeated several times—with similar questions used across different iterations—provide publicly available data on self‐reported FHC by large representative groups of men and women. One dataset, HINTS 1 (2003) has also been analyzed in one of the studies included in our literature review.^5^ However, they only included participants 50 years or older and without a personal history of colon or rectal cancer. We did not make these exclusions in our own analyses.

### 2.4 Study design and population

We used data from two public databases from surveys among noninstitutionalized US‐American civilians. The Health Information National Survey (HINTS) is a survey carried out by the National Cancer Institute aiming to gain knowledge about the use of and access to cancer information by the public.^20^ In 2003 and 2005, the survey was conducted via telephone, between 2008 and 2018 the questionnaires have been exclusively sent via mail. In 2019, an additional web‐based pilot has been introduced, where some participants had the option of filling out the questionnaire online. In one of the more recent iterations of the survey, HINTS 5 Cycle 2 (2018), the topic of special interest was FHC, and some more detailed questions about FHC were asked. We analyzed data from 10 iterations of HINTS from 2003 to 2019 in which respondents were asked about FHC.

The second large dataset we included for our analyses is the National Health Interview (NHIS).^21^ It is an annually conducted, cross‐sectional survey on a broad range of health‐related issues by the National Center for Health Statistics (NCHS) as part of the Centers for Disease Control and Prevention (CDC). NHIS data are collected through personal household interviews. We analyzed data from four iterations of NHIS from 2000 to 2015 in which respondents were asked about FHC.

### 2.5 Measures and data analyses

To assess FHC, participants of the first iteration of HINTS 1 (2003) were asked “Have any of your brothers, sisters, parents, children, or other close family members ever had cancer?’” In all later iterations of HINTS, participants were asked “Have any of your family members ever had cancer?” Participants were coded as having a positive FHC if they responded “yes” to the FHC question. In the four included iterations of the NHIS, FHC was recorded for the biological parents, biological children, and full siblings of the participant individually. For each of these relatives, participants were asked “Did your *respective relative* ever have cancer of any kind?”. Participants were coded as having a positive FHC if they indicated a positive history of cancer in any of the biological parents, biological children, or full siblings (see Table S1 for all questions and answer options, and S3 for further details).

For these FHC items, frequencies were counted using IBM SPSS Statistics 25. Then, odds ratios and confidence intervals were separately calculated for each included iteration of HINTS and NHIS. We compared the odds of men reporting a FHC with the odds of women as the reference group. In order to estimate mean gender effects individually for the HINTS and NHIS iterations as well as across both surveys, we used the metafor package (version 2.1.0) in R.^19^ Random‐effects models were fitted to the data using restricted maximum‐likelihood estimation.

In HINTS 5 Cycle 2, three additional items on FHC were asked, which we also analyzed (see below).

## 3 RESULTS

### 3.1 Review

Our literature search identified 111 records, the oldest of which dated back to 1989. After screening of titles and abstracts, 68 studies were excluded, after screening the full texts of 43 records, another 34 studies were excluded. In total, nine studies could be included in the review (see Table 1).

**TABLE 1:** Gender differences in self‐reported FHC

	Country IOC Code	N	Age range	Dataset	Relatives	FHC_Men_	FHC_Women_	OR	CI_95%_
Pinsky et al (2003)^8^	USA	149 332	55‐74	PLCO Trial	FDR	53.1	61.0	0.72	0.71‐0.74
McQueen et al (2006)^5^	USA	2686	≥50	HINTS 1	CFM	61.3	70.3	0.72	0.61‐0.85
Townsend et al (2013)^17^	USA	30 260	18‐64	2005 CHIS	FDR, SDR	28.7	34.2	0.77	0.74‐0.81
Bostean et al (2013)^14^	USA	30 520	40‐75	2009 CHIS	FDR	50.0	54.4	0.84	0.80‐0.88
Sieverding et al (2008)^7^	GER	15 810	50‐70	2004 HCAP	GP, P, S	39.1	47.9	0.70	0.66‐0.74
Hidalgo et al (2015)^15^	ESP	666	≥ 50	bespoke survey	FDR	42.3	52.7	0.66	0.48‐0.90
Hwang et al (2019)^13^	KOR	166 810	40‐79	HEXA	FDR	25.4	28.1	0.87	0.85‐0.89
Choi et al (2013)^18^	CHN	2004	≥50	bespoke survey	AFM	21.7	22.1	0.98	0.79‐1.21
Moghimi‐Dehkordi et al (2012)^16^	IRI	6453	≥20	bespoke survey	FDR, SDR	37.1	27.7	1.54	1.39‐1.71

Abbreviations: 95% CI, 95%‐Confidence Interval; AFM, Any Family Member; C, Children; CFM, Close Family Members; CHIS, California Health Interview Survey; FDR, First Degree Relatives; FHC_Men_/FHC_Women_, percentage of men/women indicating a positive Family History of Cancer (FHC); GP, Grandparents; HCAP, Health Care Access Panel; HEXA, Health Examinees Study; HINTS 1, Health Information National Trends Survey (First Iteration, 2003); N, Number of study participants; OR, Odds Ratio (men/women); P, Parents; PLCO Trial, Prostate, Lung, Colorectal and Ovarian Cancer Screening Trial; S, Siblings; SDR, Second Degree Relatives.

The included studies were published between 2003 and 2019 and provide data of self‐reported family history of any cancer in adult men and women from the US, Germany, Spain, South Korea, China, and Iran. The number of participants ranged from 666 to 166 810 summing up to a total of 404 541. The age range of participants differed between the studies (see Table 1). Four studies assessed self‐reported FHC in first‐degree relatives,^8^, ^13^, ^14^, ^15^ two studies assessed FHC in first and second‐degree relatives,^16^, ^17^ the other three studies applied other definitions of family (close family members,^5^ any family members,^18^ or grandparents, parents, or siblings^7^). Some of the surveys included only participants without a personal history of cancer.^5^, ^7^, ^8^, ^13^, ^17^ In two studies, FHC was assessed by mailed questionnaires^7^, ^8^ the other studies used telephone or personal interviews. In most studies, FHC was assessed by one question. An example is: “Have any of your brothers, sisters, parents, children, or other close family members ever had cancer?”^5^ (see Table S1 in supplemental material for an overview of FHC questions with answer options, as well as type of survey).

As can be seen in Table 1, there was substantial variance in the prevalence of self‐reported FHC between studies, the lowest rate was reported by Chinese men (21.7%), the highest rate by US women (70.3%). The odds of men reporting a positive FHC compared with women varied between 0.66 and 1.54 (see Table 1). In all studies that were conducted in the US and Europe, the odds of men reporting a positive FHC was significantly lower compared with women.^5^, ^7^, ^8^, ^14^, ^15^, ^17^ The same is true for the study from South Korea.^13^ Only two studies from Asia differed in their results: In the study from China there is no gender difference in self‐reported FHC^18^ and the study from Iran suggested higher odds of men reporting a positive FHC.^16^

The odds of men reporting a FHC is lower compared with women with an average OR of 0.84 [0.71; 1.00] across all studies. This is a significant effect, *z *= −1.99, *P* < .047, with a high heterogeneity among studies (*I* ^2^ = 99%, *Q*(8) = 322.21, *P* < .001). For the six studies from the US and Europe, the estimated average odds ratio was 0.75 [0.70; 0.80]. The effect is significant, *z* = −8.41, *P* < .001, with a high heterogeneity among studies (*I* ^2^ = 87%, *Q*(5) = 40.25, *P* < .001).

There are a number of possible methodological reasons for the large heterogeneity between studies. The studies used different definitions of family and different age groups of participants. Furthermore, the phrasing of the question with which the FHC was assessed and the type of assessment (face to face/telephone interviews, mailed questionnaires) differed between studies (see Table S1).

### 3.2 Analyses of original data

The 10 HINTS iterations included FHC data from study populations of 2728‐7185 adults with a mean age of 47.8‐57.0 years. The four NHIS iterations included study populations between 27 157 and 33 672 participants with a mean age between 46.4 and 49.9 years (see Table 2).

**TABLE 2:** Gender differences in self‐reported FHC in HINTS from 2003 to 2019 and NHIS from 2000 to 2015

	Year	N	Age_(M)_ ^a^	FHC_Men_	FHC_Women_	OR	95% CI
HINTS 1	2003	6308	47.8	58.1%	66.0%	0.71	0.64‐0.79
HINTS 2	2005	5551	52.2	68.2%	75.6%	0.69	0.61‐0.78
HINTS 3	2007	7185	54.1	68.3%	76.4%	0.67	0.60‐0.74
HINTS 4 Cycle 1	2011	3273	53.7	68.8%	74.7%	0.75	0.64‐0.87
HINTS 4 Cycle 2	2012	3240	53.8	70.2%	75.8%	0.75	0.64‐0.88
HINTS 4 Cycle 3	2013	2728	54.5	65.4%	76.9%	0.57	0.48‐0.67
HINTS 4 Cycle 4	2014	3298	55.0	69.2%	75.0%	0.75	0.64‐0.88
HINTS 5 Cycle 1	2017	2963	56.3	70.9%	77.7%	0.70	0.59‐0.83
HINTS 5 Cycle 2	2018	3121	57.0	74.2%	80.3%	0.71	0.60‐0.84
HINTS 5 Cycle 3	2019	4856	56.9	74.2%	78.5%	0.79	0.69‐0.90
*Estimated OR averaged across all HINTS iterations*						0.71	0.67‐0.74
NHIS	2000	32 374	46.4	33.3%	37.8%	0.82	0.79‐0.86
NHIS	2005	31 428	47.4	33.4%	39.0%	0.78	0.75‐0.82
NHIS	2010	27 157	47.6	33.4%	38.5%	0.80	0.76‐0.84
NHIS	2015	33 672	49.9	37.4%	40.9%	0.86	0.83‐0.90
*Estimated OR averaged across all NHIS iterations*						0.82	0.78‐0.85
*Estimated OR averaged across all HINTS and NHIS iterations*						0.75	0.71‐0.79

Abbreviations: 95% CI, 95%‐Confidence Interval; FHC_men_/ FHC_women_, Percentage of men/ women who reported a positive Family History of Cancer (FHC); HINTS, Health Information National Trends Survey; NHIS, National Health Interview Survey; OR, Odds Ratio (men/women).

^a^ Mean age of participants who reported their age.

### 3.3 Prevalence of self‐reported FHC in men and women in HINTS and NHIS data

The analyses of the data revealed prevalence rates of self‐reported FHC which differed largely between the two surveys. While in HINTS between 58.1% and 74.2% of the male participants reported a FHC, in NHIS the rates were much lower with 33.3%‐37.4%. Prevalence rates for female respondents varied between 66.0% and 80.3% in HINTS and 37.8% and 40.9% in NHIS.

The gender comparisons of self‐reported FHC, however, reveal a consistent result; across all analysed iterations of HINTS and NHIS, men consistently report less cancer in their family compared with women. The effect is more pronounced over the HINTS iterations, OR = 0.71 [0.67; 0.74]. The NHIS iterations show slightly higher odds, OR = 0.82 [0.78; 0.85]. Both effects are significant, all *P's* < .001.

The HINTS surveys show only an insubstantial amount of heterogeneity among the true effects, *I* ^2^ = 15%, *Q*(9) = 11.75, *P* = .228. For the NHIS iterations the indicator of heterogeneity shows substantial amounts of heterogeneity, *I* ^2^ = 69%, *Q*(3) = 9.81, *P* = .020.

**FIGURE 1:**
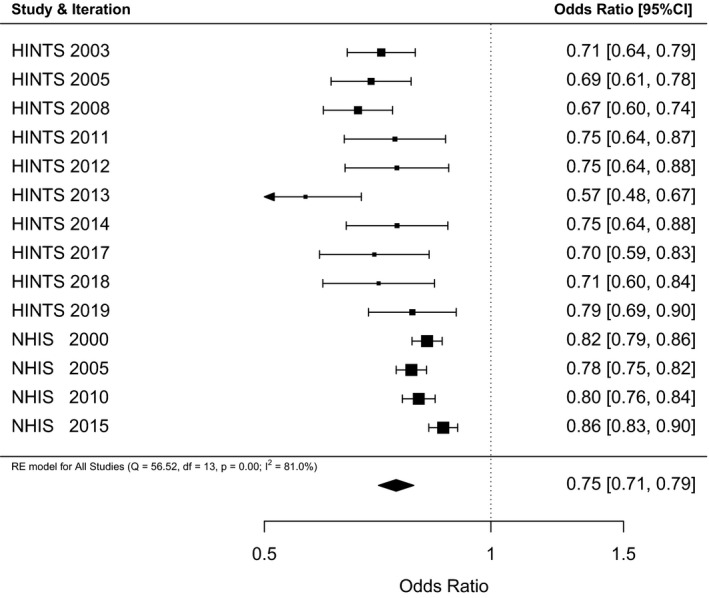
Forest plot of the odds of men reporting a positive family history of cancer (FHC) compared to the reference group of women from 10 HINTS and 4 NHIS iterations including 167 154 participants. Results are expressed as odds ratio (OR) and 95% confidence intervals

Averaged across all 10 included HINTS and 4 NHIS iterations and based on 167 154 participants, the estimated odds of men reporting a positive FHC is 0.75 [0.71; 0.79], *I* ^2^ = 81%, *Q*(13)=56.52, *P* < .001, see Figure 1.

On recommendation of a reviewer, we tested if gender differences in FHC still hold when we divide each study population of the HINTS and NHIS iterations in two age groups (<50, ≥ 50) and when controlling for personal history of cancer (PHC). The age of 50 was used as a cutoff since multiple studies included in the literature review only sampled participants equal or above this age. PHC was assessed by asking “Ever been told you had cancer?” (HINTS) and “Ever been told by a doctor you had cancer?” (HINTS). We used only clear yes and no answers.

For these analyses. we separately counted frequencies for FHC by gender for a positive PHC and negative PHC and for participants below 50 years of age and equal to or over 50 years of age. We then used Chi‐square tests to estimate separately if among participants with PHC and among participants without PHC gender differences in self‐reported FHC were shown. The same analyses were conducted for the two age groups (<50, ≥ 50). We also computed odds ratios separately for these four groups (positive PHC, negative PHC; age < 50 years, age ≥ 50 years). We compared the odds of men reporting a FHC with the odds of women as the reference group. In all NHIS and in several HINTS iterations, gender differences in FHC were no longer significant for participants with a PHC (see Table S2). However, gender differences in FHC held irrespective of age group (<50, ≥ 50) in all analyzed iterations of HINTS and NHIS (see Table S3).

### 3.4 Additional questions regarding FHC (from HINTS 5 Cycle 2)

**FIGURE 2:**
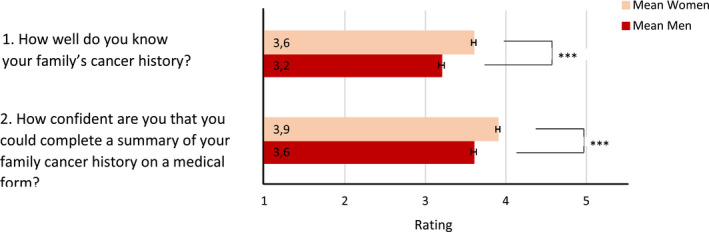
Mean ratings of additional questions regarding FHC in the HINTS 5 Cycle 2 survey (2018), Total N = 3448 (59.6% female). Error bars indicate standard error of the means. Item 1: answers from 1 (“not at all”) to 5 (“very well”). Item 2: answers from 1 (“not confident at all”) to 5 (“completely confident”). *** significant differences with *P* < .001

In HINTS 5 Cycle 2 (2018), three additional items on FHC were asked, which we also analyzed. For all of these items, family was defined as first‐ and second‐degree biological relatives such as grandparents, parents, siblings, children, aunts, uncles, nieces, and nephews. Participants were asked to indicate how familiar they are with their FHC, how confident they are that they could complete a summary of their FHC on a medical form and whether they have had discussions about their FHC with biological family members. In total 3448 participants answered these questions, of which 59.6% were female. We calculated the mean scores for the first two questions and found significant gender differences in both questions (see Figure 2). Men described themselves as less familiar with their FHC, *d* = −0.31, OR = 0.57 [0.50; 0.64] and were less confident that they could complete a summary of their FHC on a medical form, *d* = −0.30, OR = 0.59 [0.52; 0.66]. We also analyzed the proportion of men and women who stated that they had discussed their FHC with at least one family member and found a significant gender difference as well. More women (74.1%) than men (61.3%) stated that they had discussed their FHC with at least one family member, *χ* ^2^ = 63.8, *P* < .001.

## 4 DISCUSSION

The analyses presented in our manuscript reveal clear and consistent evidence for a striking gender gap in self‐reported FHC, especially in the US and in Europe. With the exception of two studies from China and Iran, men are consistently less likely to report a FHC than women. We do not know the true prevalence of FHC in the male and female samples that were included in our analyses, but given the consistency and size of the gender differences suggest that men in these studies on average underreported FHC.

Our interpretation is consistent with findings from other research. For example, Pinsky and colleagues (2003) interpreted the identified substantial gender gap in self‐reported FHC in their study as follows: “In the current study, it seems likely that most of the differential was made up by males’ omitting cancer events rather than by females inventing them.”^8^ Overreporting of FHC has been shown to be generally low^22^ and studies that assessed self‐reported FHC in relatives of persons with confirmed diagnoses found evidence for underreporting in both sexes. The degree of concordance between diagnoses reported by the family and confirmed diagnoses was between 72% in 1363 breast cancer families and 77% in 764 colorectal cancer families.^23^ Another study that assessed self‐reports of FHC of colorectal cancer among first‐degree relatives of colorectal cancer patients revealed that even in this special sample, about a quarter of respondents reported having *no* first‐degree relative with colon cancer. Males were significantly less aware of the case of (colorectal) cancer in their family compared with females.^24^

What could be the reasons for the gender gap in self‐reported FHC? Cancer‐specific differences in reporting biases might offer indirect evidence. Several studies have found that self‐reported FHC is most accurate for breast cancer.^22^, ^25^, ^26^, ^27^ Conversely, Mai and colleagues (2011) found that survey respondents with a family history of prostate cancer were most likely to falsely report no cancer in their family.^25^ These site‐specific differences suggest that women may be the family disseminator of health information,^28^, ^29^ while men are less willing to discuss health issues or disseminate health information leading to low awareness among relatives of this cancer diagnosis.^25^ Our own analyses of data from the 2013 HINTS 5 Cycle 2 iteration revealed that compared with women fewer men discussed FHC with other family members, men described themselves as less familiar with one's FHC, and as less confident in filling out medical forms about FHC. In line with these findings are the results from the US Health Styles Survey which reported that men are less likely (23%) compared with women (36%) to actively collect health information on their relatives’ health family history.^30^ A recent study also using data from the HINTS 4 Cycle 3 (2013) iteration indicated that those (altogether about one‐third) participants who believed that family health history was not important for their health were significantly more likely to be male (57%) than female (43%).^31^

Traditional masculinity norms which expect men to be independent and emotionally controlled and regard psychological or medical help‐seeking as “weak” or “unmanly” were identified as factors associated with poor communication with health professionals. They also explained delays in medical help‐seeking in men in general^32^ as well as men's help‐seeking for cancer symptoms.^33^ Identification with traditional masculinity norms could be a barrier to talking about and reflecting on FHC. A study that investigated information dissemination in families with a high risk of (breast) cancer reported that women tend to be information gatherers or disseminators, whereas men tend to be information blockers.^28^

For all NHIS and several HINTS iterations we did not find gender differences in self‐reports of FHC for participants with a personal history of cancer. This could be due to the fact that at last men talk about FHC when they are faced with a cancer diagnosis. Mitchell and colleagues (2013) for instance reported PHC as a highly significant predictor for African American men discussing FHC with their families.^34^ In addition, a cancer diagnosis comes along with visits to many different physicians who usually ask about the patient's FHC. Flynn and colleagues (2010) questioned several hundred US physicians about their use of FHC. About 96% of them reported always taking information at least on parents’ FHC.^35^ The more heterogeneous findings for the HINTS iterations in comparison to the clear picture seen in the NHIS iterations might arise from smaller sample sizes of the HINTS data.

What could be the reasons for the large variations in the prevalence of self‐reported FHC? We found large differences in the self‐reports of the overall prevalence of FHC across studies not only in the reviewed studies from different countries but in the analyses of US data from HINTS and NHIS. While roughly 72% of participants reported a positive FHC in HINTS, only 37% of participants did so in NHIS. One plausible explanation might be the question format used for assessing FHC. Asking for an overall FHC across all relatives as it was done since HINTS 2 seems to lead to a fairly high prevalence, whereas asking for specific relatives as it was done in NHIS seems to lead to lower prevalence. This argument becomes more pertinent, looking at some striking differences in prevalence which we identified. Firstly, there is a rise of roughly 10% between HINTS 1 and subsequent HINTS iterations (see Table 2). Interestingly, in HINTS 1, a more specific question had been asked, naming brothers, sisters, parents, children, or other close family members as the reference group. Subsequently, the question was kept more general, just asking whether any family members had ever had cancer. The second difference in prevalence that stands out, is between the studies of Townsend et al (2013)^17^ and Bostean et al (2013)^14^ in Table 1. Both used data from different iterations of the California Health Interview Survey (CHIS), which is a regularly conducted large health survey in California, similar to HINTS and NHIS. While in 2005, individual questions were asked for specific groups of relatives, in 2009 CHIS only asked one question on overall FHC. Even though the individual questions from 2005 also included second‐degree relatives, the prevalence was lower compared with CHIS 2009 results which only asked about first‐degree relatives. Future research should investigate which question format is better able to increase the accuracy of FHC reporting.

### 4.1 Strengths and limitations

Our analyses are based on large datasets from population‐based studies from different countries and especially on several iterations of two representative US‐American Health Surveys. The gender gap in self‐reported FHC we identified is very consistent across studies and across different ways of assessing FHC.

However, we are unable to pinpoint the exact reasons for the gender differences found. The methods used by the included studies are unable to discriminate between a lower awareness and an equal awareness but a lower likelihood of reporting.

It should also be noted that most studies that were identified by our literature search were conducted in the US or in Western countries. One large study from South Korea showed the same pattern, whereas two studies from China and Iran revealed no gender effect and a reversed gender effect. Our conclusion that men underreport FHC is therefore limited to the situation in Western countries and especially to the situation in the US. Another limitation is the fact that we cannot compare self‐reported FHC with true prevalence data.

A further limitation lies in the FHC data we analyzed from the NHIS surveys. Due to the different format of assessing FHC in separate relatives we calculated the variable self‐reported FHC of any cancer as it was done in prior research using NHIS data.^6^ The format which is used to assess FHC in NHIS is more detailed than in HINTS but also more difficult to interpret because FHC is assessed for several family members individually. This difficulty of merging the answers of the detailed questions into one FHC score might be the reason why a recent study which analyzed NHIS 2015 data did not include FHC as a predictor for genetic counseling.^36^

### 4.2 Implications for further research and medical practice

The average odds of men reporting a positive FHC compared with women was lower, with 0.71, in the HINTS surveys compared with 0.82 in the NHIS surveys. In the HINTS surveys participants filled in questionnaires, whereas in the NHIS surveys participants were personally interviewed. Future research should investigate the question of whether questionnaires result in larger gender gaps in self‐reported FHC compared to personal interviews and how the assessment of FHC can be improved to reduce gender inequalities in reporting.

Furthermore, future studies should investigate whether the gender difference holds for all or only certain cancer subtypes, as we only investigated the overall family history of cancer.

When assessing FHC in clinical practice and research, the problem of potential underreporting among men should be kept in mind. The importance of FHC is likely to increase with the expansion of risk‐stratified cancer screening and genetic counseling. Biased reporting of FHC can lead to important inequalities in health care access and subsequent increasing inequalities in cancer outcomes. Health care professionals should help men to reconstruct their FHC with open questions instead of dichotomous interviewing and providing men with the necessary information on why knowledge about FHC is important and how FHC can be recollected.

## CONFLICT OF INTEREST

The authors have no conflicts of interest to declare.

## AUTHOR CONTRIBUTIONS

Monika Sieverding: Conceptualization of the research idea, designing the search strategy and conducting the literature search, study selection for the review, survey selection for the secondary analyses, writing original draft and writing review and editing, supervision. Lisa Arbogast: Study selection for the review, survey selection for the secondary analyses, processing the data for the secondary analyses, conducting the statistical analyses, aiding MS in writing the original draft, writing review and editing. Stephanie Zintel: Conducting the statistical analyses and visualizing data. Christian von Wagner: Conceptualization of the research idea, designing the search strategy, survey selection for the secondary analyses, writing review and editing. All authors approved the final version and assisted with revisions.

## Supporting information

Supplementary Material

## Data Availability

The data that support the findings of this study are available from the corresponding author upon reasonable request.
