# Supplementary material for: Gender differences in self‐reported family history of cancer: A review and secondary data analysis
Source: Cancer Med. 2020 Aug 24;9(20):7772–80. doi: 10.1002/cam4.3405 (PMC7571831; doi:10.1002/cam4.3405)
Supplement: Supplementary file 1 — Supplementary Material [file CAM4-9-7772-s001.docx]

**Supplementary Material Table of Contents**

**Supplement 1:** Search Strategy
**Supplement 2:** Inclusion and Exclusion Criteria
**Figure S1:** Flowchart of Study Selection
**Table S1:** FHC item wording of included studies and data sets
**Table S2:** Gender Differences in Self-Reported FHC in HINTS from 2003 to 2019 and NHIS from 2000 to 2015 split by Personal History of Cancer (PHC)
**Table S3:** Gender Differences in Self-Reported FHC in HINTS from 2003 to 2019 and NHIS from 2000 to 2015 split by Age
**Supplement 3:** Coding and analysis of FHC items
**References**

**Supplement 1: Search Strategy**

Electronic database searches were conducted on Web of Science and PubMed using the following keywords:

**ALL FIELDS:** ("family history of cancer")

*AND* **ALL FIELDS:** (questionnaire or survey or interview or cross-sectional)

*AND* **ALL FIELDS:** (women and men or female* and male* or gender or sex)

*NOT* **ALL FIELDS:** (case report or clinical study or randomized trial or case-control)

All searches were done on the 20th of November 2019. They resulted in 59 hits on Web of Science and 70 hits on PubMed.

The electronic searches were supplemented by manual searches in relevant studies and bibliographies that were identified in a prior literature search by MS and CvW.

**Supplement 2: Inclusion and Exclusion Criteria**

**Inclusion Criteria**

1. Cross-sectional, quantitative data
2. Population-based community samples of healthy adults
3. Provision of frequency statistics about self-reported FHC for any cancer for both men and women

**Exclusion Criteria**

1. Studies based on specific subgroups within the population
   (such as patients, smokers, relatives of cancer patients, African Americans, immigrants, people with physical or mental disabilities, students, rural population, health care professionals)
2. Qualitative studies
3. Case reports
4. Case-control studies
5. Cohort studies (exception: baseline assessment)
6. Longitudinal studies (exception: baseline assessment)
7. Publications in languages other than English or German

11 additional studies identified through manual searches

34 studies excluded

18 no gender-differentiated FHC data

8 inadequate samples

6 data on FH of a specific cancer

2 otherwise inadequate recording FH data

29 duplicates removed

68 studies excluded

129 studies identified from electronic databases

59 Web of Science

70 PubMed

111 titles and abstracts screened

9 studies included in analyses

43 full texts assessed for eligibility

***Figure S1*:** Flowchart for Study Selection

***Table S1:* Exact wording of Family History of Cancer items in included studies and data sets**

|  | Item(s) | Answer Options | Type of Survey |
| --- | --- | --- | --- |
| Pinksy et al. (2003)^1^: PLCO Trial | Have your parents, children, brothers, sisters, half-brothers, or half-sisters ever been diagnosed as having any type of cancer? (Do not include basal-cell skin cancer) | No, yes | Questionnaire |
| McQueen et al. (2006)^2^: HINTS 1 | see HINTS 1 | see HINTS 1 | see HINTS 1 |
| Townsend et al. (2013)^3^: CHIS 2005 | These next questions ask about your FHC. By family we mean only your blood relatives, including half brothers and sisters:   - Have any of your grandparents ever had cancer of any kind? - Has your father or mother, or have any of your brothers, sisters, sons or daughters ever had cancer of any kind? - Have any of your parents’ brothers or sisters, that is, your uncles or aunts, ever had cancer of any kind? | Yes, no, refused, don’t know | Telephone Interview in Household |
| Bostean et al. (2013)^4^: CHIS 2009 | Now I’m going to ask about your FHC. By family, we mean only your blood relatives. Did your biological father or mother, full brothers or sisters, or biological sons or daughters ever have cancer of any kind? | Yes, no, refused, don’t know | Telephone Interview in Household |
| Sieverding et al. (2008)^5^: HCAP 2004 | *Translation from German:* Are your grandparents, parents or siblings known to have cancer? | No; yes, one person; yes, 2 or more persons | Questionnaire via Mail |
| Hidalgo et al. (2015)^6^: bespoke data | not obtainable | not obtainable | Personal Interview |
| Hwang et al. (2019)^7^: HEXA | Among your first-degree relatives including parents, siblings, or children, is there any member who has been diagnosed with cancer by a physician? | Yes, no | Personal Interview (interview-based questionnaire) |
| Choi et al. (2013)^8^: bespoke data | not obtainable | not obtainable | Telephone Interview |
| Moghimi-Dehkordi et al. (2012)^9^: bespoke data | not obtainable | not obtainable | Personal Interview |
| HINTS 1 (2003) | Have any of your brothers, sisters, parents, children, or other close family members ever had cancer? | Yes, no, no family, refused, don’t know | Telephone Interview |
| HINTS 2 – 3 (2005, 2008) | Have any of your family members ever had cancer? | Yes, no, no family, refused, don’t know | Telephone Interview (HINTS 2 & 3), Questionnaire via Mail (HINTS 3) |
| HINTS 4 – 5 (2011 – 2019) | Have any of your family members ever had cancer? | Yes, no, not sure | Questionnaire via Mail, Online Questionnaire (HINTS 5 Cycle 3) |
| NHIS (2015, 2010, 2005, 2000) | We would like to ask you a few questions about your FHC:   1. Did your *biological mother/father* ever have cancer of any kind? 2. Did your *biological daughter(s)/biological son(s)/full brother(s)/full sister(s)* ever have cancer of any kind? How many of your *biological daughter(s)/biological son(s)/full brother(s)/full sister(s)* have ever had cancer of any kind? | 1. Yes, no, adopted or don’t know biological father/mother, refused, don’t know 2. number of daughters/sons/ brothers/sisters, refused, don’t know | Personal Interview in Household |

FHC: Family History of Cancer

*Table S2:*

**Gender Differences in Self-Reported FHC in HINTS from 2003 to 2019 and NHIS from 2000 to 2015 split by Personal History of Cancer (PHC)**

|  | **PHC** | **N** | $\boldsymbol{\chi}^{\boldsymbol{2}}$ **(1)** | **p** | **OR [CI]** |
| --- | --- | --- | --- | --- | --- |
| HINTS 1 | Yes | 753 | 1.516 | .218 | 0.81 [0.58;1.13] |
|  | No | 5540 | 35.856 | <.001 | 0.71 [0.64;0.80] |
| HINTS 2 | Yes | 865 | 8.359 | .004 | 0.62 [0.45;0.86] |
|  | No | 4634 | 26.931 | <.001 | 0.70 [0.62;0.80] |
| HINTS 3 | Yes | 976 | 12.016 | .001 | 0.59 [0.44;0.80] |
|  | No | 6194 | 46.231 | <.001 | 0.67 [0.60;0.76] |
| HINTS 4 Cycle 1 | Yes | 501 | 3.840 | .050 | 0.65 [0.42;1.00] |
|  | No | 2772 | 10.668 | .001 | 0.76 [0.64;0.90] |
| HINTS 4 Cycle 2 | Yes | 433 | 0.181 | .670 | 0.90 [0.54;1.49] |
|  | No | 2805 | 14.497 | <.001 | 0.72 [0.61;0.85] |
| HINTS 4 Cycle 3 | Yes | 405 | 12.665 | <.001 | 0.38 [0.22;0.66] |
|  | No | 2323 | 31.941 | <.001 | 0.59 [0.50;0.71] |
| HINTS 4 Cycle 4 | Yes | 480 | 4.955 | .026 | 0.59 [0.37;0.94] |
|  | No | 2813 | 9.212 | .002 | 0.77 [0.66;0.91] |
| HINTS 5 Cycle 1 | Yes | 444 | 3.719 | .054 | 0.61 [0.36;1.01] |
|  | No | 2514 | 13.244 | <.001 | 0.72 [0.60;0.86] |
| HINTS 5 Cycle 2 | Yes | 539 | 0.277 | .598 | 1.15 [0.69;1.90] |
|  | No | 2582 | 20.227 | <.001 | 0.66 [0.55;0.79] |
| HINTS 5 Cycle 3 | Yes | 770 | 2.668 | .102 | 0.71 [0.47;1.07] |
|  | No | 4086 | 11.028 | .001 | 0.79[0.68;0,91] |
| NHIS 2000 | Yes | 2151 | 1.025 | .311 | 1.10 [0.92;1.31] |
|  | No | 30195 | 63.976 | <.001 | 0.82 [0.78;0.86] |
| NHIS 2005 | Yes | 2428 | 0.117 | .733 | 0.97 [0.82;1.15] |
|  | No | 28969 | 99.166 | <.001 | 0.78 [0.74;0.82] |
| NHIS 2010 | Yes | 2333 | 0.428 | .513 | 0.94 [0.80-1.12] |
|  | No | 24804 | 65.665 | <.001 | 0.80 [0.76;0.85] |
| NHIS 2015 | Yes | 3289 | 0.009 | .926 | 1.01 [0.87;1.16] |
|  | No | 30350 | 40.716 | .001 | 0.86 [0.82;0.90] |

OR = Odds Ratio (men/women). CI = 95%-Confidence Interval

*Table S3:*

**Gender Differences in Self-Reported FHC in HINTS from 2003 to 2019 and NHIS from 2000 to 2015 split by Age**

|  | **Age** | **N** | $\boldsymbol{\chi}^{\boldsymbol{2}}$ **(1)** | **p** | **OR [CI]** |
| --- | --- | --- | --- | --- | --- |
| HINTS 1 | <50 | 3567 | 22.638 | <.001 | 0.72 [0.63;0.82] |
|  | ≥50 | 2716 | 13.577 | <.001 | 0.74 [0.62;0.87] |
| HINTS 2 | <50 | 2509 | 11.149 | .001 | 0.74 [0.62;0.88] |
|  | ≥50 | 3024 | 22.565 | <.001 | 0.67 [0.56;0.79] |
| HINTS 3 | <50 | 2759 | 14.901 | <.001 | 0.72 [0.60;0.85] |
|  | ≥50 | 4380 | 43.37 | <.001 | 0.63 [0.55;0.73] |
| HINTS 4 Cycle 1 | <50 | 1273 | 4.239 | .040 | 0.77 [0.60;0.99] |
|  | ≥50 | 1960 | 12.488 | <.001 | 0.69 [0.57;0.85] |
| HINTS 4 Cycle 2 | <50 | 1256 | 6.904 | .009 | 0.71 [0.55;0.92] |
|  | ≥50 | 1947 | 6.631 | .010 | 0.76 [0.62;0.94] |
| HINTS 4 Cycle 3 | <50 | 988 | 15.068 | <.001 | 0.57 [0.43;0.76] |
|  | ≥50 | 1688 | 27.834 | <.001 | 0.56 [0.45;0.69) |
| HINTS 4 Cycle 4 | <50 | 1136 | 4.861 | .027 | 0.74 [0.57;0.97] |
|  | ≥50 | 2104 | 9.235 | .002 | 0.74 [0.60;0.90] |
| HINTS 5 Cycle 1 | <50 | 949 | 3.968 | .046 | 0.74 [0.55;1.00) |
|  | ≥50 | 1974 | 17.118 | <.001 | 0.65 [0.52;0.79] |
| HINTS 5 Cycle 2 | <50 | 955 | 10.416 | .001 | 0.61 [0.45;0.83] |
|  | ≥50 | 2121 | 7.932 | .005 | 0.74 [0.60;0.91] |
| HINTS 5 Cycle 3 | <50 | 1497 | 1.067 | .302 | 0.88 [0.69;1.12] |
|  | ≥50 | 3297 | 13.911 | <.001 | 0.73 [0.62;0.86] |
| NHIS 2000 | <50 | 19697 | 29.327 | <.001 | 0.84 [0.79;0.89] |
|  | ≥50 | 12677 | 21.728 | <.001 | 0.85 [0.79;0.91] |
| NHIS 2005 | <50 | 17948 | 42.702 | <.001 | 0.80 [0.75;0.85] |
|  | ≥50 | 13480 | 46.844 | <.001 | 0.79 [0.74;0.84] |
| NHIS 2010 | <50 | 15020 | 18.706 | <.001 | 0.85 [0.78;0.91] |
|  | ≥50 | 12137 | 35.768 | <.001 | 0.80 [0.75;0.86] |
| NHIS 2015 | <50 | 16616 | 7.334 | .007 | 0.91 [0.84;0.97] |
|  | ≥50 | 17056 | 23.067 | <.001 | 0.86 [0.81;0.92] |

OR = Odds Ratio (men/women). CI = 95%-Confidence Interval

**Supplement 3:** Coding and analysis of FHC items

Table S1 illustrates the wording and answer options in all HINTS iterations. Following McQueen et al. (2006)^2^ we only considered participants clearly answering “yes” or “no” to the FHC item for the current analyses. All participants giving other answers such as “don’t know”, “not sure”, or “no family” (depending on the iteration), participants with missing data or participants giving multiple responses in error were not included. Therefore, the sample sizes in our analyses are somewhat smaller than the number of participants in the respective HINTS iterations.

As Table S1 shows, NHIS assessed FHC differently. Participants were asked to give a detailed FHC for specific relatives individually. To compare NHIS data to the publications included in our review, as well as HINTS data we calculated an overall FHC measure. Participants were coded as having a positive FHC if they indicated a positive FHC in any of the biological parents, biological children or full siblings as it was done in an earlier study analysing NHIS data.^10^ All other responses were considered a negative FHC. This included answering no, don’t know, adopted, don’t know biological father/mother or any combinations of these answers. Unlike in HINTS, we could not just extract data from people who consistently answered no due to the variability in responses which would have meant only including just over 25% of responders. This approach is also in line with a previous publication by Scheuner, McNeel & Freedman (2010).^11^ In addition to “no” they also classified “I don’t know” and missing responses (refused, not ascertained) as a negative FHC in their analyses of a large Californian interview study.

**References**

1. Pinsky PF, Kramer BS, Reding D, Buys S. Reported family history of cancer in the prostate, lung, colorectal, and ovarian cancer screening trial*.* *Am J Epidemiol* 2003; **157**: 792–99.
2. McQueen A, Vernon SW, Meissner HI, Klabunde CN, Rakowski W. Are there gender differences in colorectal cancer test use prevalence and correlates? *Cancer Epidemiol Biomarkers Prev* 2006; **15**: 782–91.
3. Townsend JS, Steele CB, Richardson LC, Stewart SL. Health behaviors and cancer screening among Californians with a family history of cancer*.* *Genetics in Medicine* 2013; **15**: 212–21.
4. Bostean G, Crespi CM, McCarthy WJ. Associations among family history of cancer, cancer screening and lifestyle behaviors: A population-based study. *Cancer Causes Control* 2013; **24**: 1491–1503.
5. Sieverding M, Matterne U, Ciccarello L. Gender differences in FOBT use: Evidence from a large German survey*.* *Z Gastroenterol* 2008; **46**: 47–51.
6. Hidalgo JLT, Sotos JR, Herráez MJS, Rosa MC, López JLT, Ortiz MPS. Factors Associated With Cancer Worry Among People Aged 50 or Older, Spain, 2012-2014*.* *Preventing Chronic Disease* 2015; **12:** e226.
7. Hwang MJ, Zhang HS, Park B. Association between health behaviors and family history of cancer according to sex in the general population*.* *Am J Prev Med* 2019; **56**: 393–403.
8. Choi KC, So WK, Chan DN, et al. Gender differences in the use of colorectal cancer tests among older Chinese adults. *European Journal of Oncology Nursing* 2013; **17**: 603–9.
9. Moghimi-Dehkordi B, Safaee A, Vahedi M, Pourhoseingholi MA, Pourhoseingholi A, Ashtari S. History of upper gastrointestinal cancers in relatives: A community-based estimate*.* *Gastroenterology and Hepatology from Bed to Bench* 2012; **5**: 100–5.
10. Ramsey SD, Yoon P, Moonesinghe R, Khoury MJ. Population-based study of the prevalence of family history of cancer: Implications for cancer screening and prevention. *Genetics in Medicine* 2006; **8**: 571­­­−75.
11. Scheuner MT, McNeel TS, Freedman AN. Population prevalence of familial cancer and common hereditary cancer syndromes. The 2005 California Health Interview Survey*.* *Genetics in Medicine* 2010; **12**: 726–35.
